# Precision and Virtual Care

**DOI:** 10.1055/s-0044-1800717

**Published:** 2025-04-08

**Authors:** Elizabeth M. Borycki, Femke van Sinderen, Linda Dusseljee Peute, Sasha Zinovich, David Kaufman, Vivian Vimarlund, Andre W. Kushniruk

**Affiliations:** 1 School of Health Information Science, University of Victoria, Victoria, Canada; 2 Amsterdam University Medical Centers, Amsterdam, Netherlands; 3 SUNY Downstate, Health Sciences University, New York, United States of America; 4 Department of Computer and Information Science (IDA), School of Engineering and Technology, Linköping University, Sweden

## Abstract

The importance of virtual care has been highlighted by the recent pandemic which emphasized the need for effectively providing care remotely. In addition, the development of a range of emerging technologies to support virtual care has accelerated this trend. Technologies may vary in complexity from low (e.g., technologies that can be used easily by patients) to high (e.g., use of sophisticated software and hardware to support virtual care). In this article virtual care is first defined, followed by a discussion of a range of virtual care technologies. A framework is then described that can be used to consider and reason about virtual care in terms of both technology complexity as well as patient complexity. Examples of virtual care that can be considered using the framework are provided. It is argued that achieving an appropriate fit between the level of complexity of the technology involved and patient context will lead to improved care and ultimately precision virtual care. Implications of the approach presented are explored.

## 1. Introduction

The recent COVID-19 pandemic highlighted the importance of countries being able to provide virtual care to immunocompromised and chronically ill children and older adults, who were at greater risk of death, hospitalization and long-term symptoms of the disease [ 1 , 2 ]. Virtual care has also protected health professionals from unnecessary exposure to COVID-19 at the height of the pandemic, when there was an increased demand for healthcare as the number of individuals in the general population who contracted the disease increased. During this period, shortages of personal protective equipment emerged and illness among health professionals, who were treating patients, lead to a need for health professionals to self-isolate and receive medical care at the same time [ 3 , 4 ].

Today, virtual care and the technologies that support health services have become ubiquitous in their use internationally, with the range of low through to high income countries using similar technologies to provide these services [ 5 ]. Virtual care has provided citizens with improved access to physicians in regions, where there are shortages of doctors and other health professionals. Virtual care is also being touted as a means of reducing fossil fuel use associated with unnecessary health-related travel [ 6 ]. Yet, even as virtual care has accelerated in its use by citizens and health professionals, there is a need to consider the broad nature of these emerging technologies. This includes their use in terms of their fit with the health needs of citizens and the work context, where health professionals provide care [ 7 8 9 10 ].

Technology fit with citizen and health professional digital ecosystems will influence the effectiveness and efficiency associated with the use of virtual care technologies in the long term. There will be a need to attend to technology fit in the context of citizen wellness and complexity of disease to ensure a precise fit is achieved between virtual care and citizen health needs [ 10 ]. This will be necessary to avoid costly, inappropriate, and unsafe use of virtual care technologies [ 7 , 8 , 11 ]. In this paper we outline a framework for considering the selection, implementation and use of technologies that comprise virtual care. We take the perspective that for virtual care technologies to be effective, efficient, safe, and low cost, there is a need for greater attention to precision in the selection, implementation and use of virtual care by healthcare organizations and country healthcare systems of digital care. Precision in the selection, implementation, and use of virtual care will account for complexity associated with health-related considerations for citizens (and patients) regarding wellness, diseases, and conditions. Therefore, to achieve technology fit with citizen health needs and health professionals work context the concept of precision or tailored virtual care will need to be considered into the future.

The objectives of this paper are five fold:

- To define virtual care and provide a brief description of some of the technologies that are now considered to be part of this type of care;
- To provide a definition for virtual care technology fit, complexity in the context of citizen disease and health conditions, as well as precision healthcare;
- To describe a framework to support the understanding of the relation between virtual care technology fit, citizen disease, citizen health conditions, and precision;
- To provide illustrative examples of virtual care technologies that would need to be considered from a citizen health and precision perspective and how there needs to be consideration of this from a digital health ecosystem perspective;
- To identify future research directions.

## 2. Defining Virtual Care

Virtual care has been defined in many ways [ 7 , 8 , 12 ]. Prior to the pandemic, organizations such as the World Health Organization used terms such as telehealth, telemedicine and mHealth [ 8 ]. During the pandemic, we saw the emergence of the term virtual care. Virtual care definitions drew on the telehealth and telemedicine literature. For example, in 2018 virtual care was defined “as any interaction between patients and/or members of their circle of care, occurring remotely, using any forms of communication or information technologies with the aim of facilitating or maximizing the quality and effectiveness of patient care” [ 12 ]. With the advent of COVID-19 and the announcement of a global pandemic, virtual care emerged as a solution to providing access to healthcare while protecting health professionals and vulnerable citizens or patients from contracting the disease [ 1 , 3 , 4 ]. Pressures emerging from the pandemic led to the expansion of the definition of the term beyond video conference calls and mobile health applications [ 4 , 5 , 12 ] to include communication technologies, information technologies, medical devices, sensors and consumer technologies that can be used to provide health and wellness services [ 7 8 9 , 11 ] (see Table 1 ).

**Table 1.** Examples of Existing and Emerging Virtual Care Technologies.

Patient portals Blood pressure monitoring devices Electronic health record Temperature monitoring devices Oxygen monitoring devices Video conferencing tools Virtual reality Wireless networks

In Table 1 existing, new and emerging technologies form the basis for virtual care. Technologies can be used individually or in combination with other technology tools to provide virtual care. To illustrate, electronic health records could be used in conjunction with video conferencing tools by a physician to review laboratory results with the patient via video conference call. Here, the doctor could share the screen displaying the laboratory results. The patient or citizen could share information recorded in their mobile application about diet and exercise with the physician by communicating this information or sharing it using the screen sharing function that is part of the video conferencing tool. Existing technology such as the electronic health record in conjunction with other technologies such as medical devices, sensors and video conferencing are part of virtual care.

Consequently, post pandemic virtual care has been re-defined as the technologies and models of care that can be used to provide healthcare to citizens virtually. As this list of technologies is continuing to emerge, expand and grow so will virtual care [ 8 ]. Today, virtual care can be defined as the provision of care that encompasses citizens, health professionals', healthcare organizations and healthcare digital ecosystems. Virtual care includes all technologies used to provide care in varying contexts, where some part of the digital health ecosystem is virtualized. This may include electronic health records, patient portals, mobile applications used for health, medical devices, video conference calls and other emerging technologies applied to health that are unique to each citizen's health needs and conditions. Ideally, virtual care should be provided using a precision approach with the technologies matching the citizen's or patient's needs. Virtual care technologies should be tailored to the citizens (or patient's) diseases, health conditions and the work context of the user (e.g., doctor, nurse practitioner, nurse, physiotherapist).

## 3. Virtual Care, Technology-fit, Complexity and Precision

### 3.1. Virtual Care

Today, virtual care is comprised of software, computer hardware, medical devices, consumer technologies, and networking technologies [ 7 , 8 ]. Citizens can be well, diagnosed with a single disease, or they can have multiple diseases (i.e., a citizen can have multiple co-morbidities) [ 13 ]. In addition to this, citizen's may have several health conditions that may affect physical function (i.e., activities of daily living and instrumental activities of daily living) and their cognition (i.e., their ability to think and reason so that they can live in the community independently and self-manage their health) [ 14 ].

Today, varying technology types and configurations are increasingly being used to help citizens (or patients) live at home and engage with society [ 7 , 8 , 15 ]. The complexity of these technologies is growing as new digital innovations are being added and others become obsolete and are replaced [ 16 ]. To illustrate, electronic health records allow health professionals to review citizen information during video visits that are generated from the electronic health record (i.e., the doctor clicks on a button to initiate a virtual care appointment with a patient). During the healthcare visit, the citizen may provide verbal information, send photos, or even demonstrate the problem they are encountering (e.g., demonstrating a limited range of motion of a knee for the health professional to review) during the video call. In the example above, several technologies are used to provide virtual care. Identifying the types of technologies that need to be used and their configuration of use has now emerged as a critical aspect of providing virtual care. There is a need to assess for additional technologies that would enable a successful health visit. Such knowledge is now essential for citizens to receive effective, efficient, and safe care that is specific to their health and the disease(s) they may be diagnosed with [ 7 ].

It must be noted that even as the health professionals and citizens use these technologies, other new digital health technologies may be implemented that may add to the quality and safety of the virtual care visit by improving the number and types of information that are used to help the clinician develop a robust, situationally aware, patient mental model [ 17 , 18 ]. For example, if a country or county/province/state implements a patient portal [ 19 20 21 ] or the citizen begins a new physiotherapy regime that is incorporated into a digital technology such as a video game-based form of physiotherapy [ 21 22 23 ], the health professional may review the results of the patient's video game-based physiotherapy via data that was uploaded to the electronic health record. Such information will inform clinician decision making and help the health professional to identify new technology configurations (i.e., of the video game-based physiotherapy program) that will better motivate the citizen (i.e., patient) and improve their physical function and performance. Patient rehabilitation outcomes and their subsequent self-management and independence will improve as a result [ 19 20 21 ].

### 3.2. Technology-fit

Technology-fit is an important aspect of virtual care [ 12 ]. Like other technologies there is a need to create a seamless cognitive socio-technical fit between the technology, citizen, and health professionals' cognitive aspects of work (i.e., information seeking, reasoning and decision making) and sociologic contexts of healthcare [ 17 , 18 , 24 ]. We know from prior research that good fit is crucial to improving the quality and safety of care [ 25 ]. Poor fit leads to potential missed diagnoses or mis-diagnosis(es) [ 26 , 27 ]. Poor fit may also lead to cumbersome, unwieldy workarounds that may introduce additional taks and new types of errors arising from interactions between the individual, technology, and the environmental context. Unwieldy workarounds may also lead to information losses that may have better informed health professional decision making [ 28 ]. As the number of potential technologies that can be used as part of virtual care grows, both in the public and private sector, there will be a greater need to attend to fit and develop new models of interaction between technology innovators, researchers, implementers, vendors, and customers (i.e., citizens, health professionals and health focused organizations) [ 29 ]. This growing area of research has begun to focus on aspects of cognitive socio-technical fit to create precision (or tailored) virtual care for the patient.

### 3.3. Complexity and Precision

The selection, procurement, implementation and use of varying technologies and their associated configurations for patient or citizen health will be critical to achieving cognitive socio-technical fit and precision healthcare, which is personalized and tailored to each patient's or citizen's health needs and contexts. Emerging and existing technology innovations need to be reviewed within from a user(s) perspective. Citizen and health professional cognitive socio-technical fit will affect selection of virtual care technologies, and this will in turn influence wellness activities, disease trajectories, stage of disease, cognitive capacity, and physical function. When the above-mentioned is considered precision virtual care will be achieved over a citizen's life span.

## 4. A Framework for Understanding Virtual Care Technology Fit, Citizen Healthcare, and Precision

**Figure 1.**
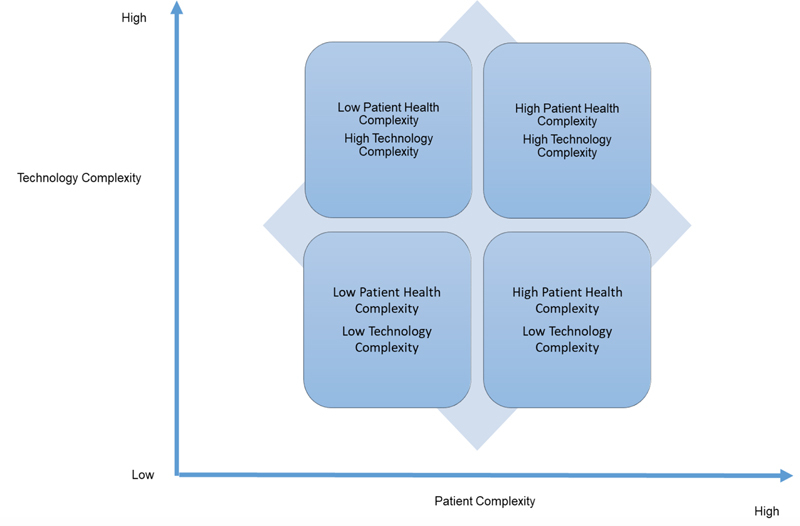
Citizen (and Patient) Heath Complexities and Virtual Care.

Precision virtual care is now acting as a virtual care innovation driver for individual technologies and the design of digital health ecosystems of care (see Figure 1 ). Today, technology and patient (or citizen) health complexity are drivers of healthcare processes and there will be a need to continue viewing this work through a precision lens to achieve cognitive socio-technical fit and to achieve value through precision virtual care. This will be necessary as everyone is unique and their expression of health conditions and disease is unique in their context of work, community, and individual life [ 30 ]. One can view this using a framework, where citizens have varying complexities in their health needs and technology, if viewed from a precision lens [ 31 32 33 ] (see Figure 1 ).

Figure 1 is a framework that can help health informatics professionals to reason about citizen health complexities and how precision (tailored) virtual care technologies can be used to support them. In Figure 1 on the x axis, patient's range in their health complexity from high to low. This is driven by key aspects of their health, including type of health condition, stage of disease, trajectory of disease, and the presence or absence of other co-morbidities, which add to health management complexity for citizens and health professionals. In addition to this, cognitive and physical function may add to the complex nature of health management; for example, a cognitive deficit or an inability to perform activities of daily living (ADL's) or instrumental activities of daily living (IADL's) may make health management more complex for the health professional. This might require the addition of one or more technologies and their implementation will need to fit the needs of the individual citizens precisely to afford improvements in patient health outcomes. Fit with the citizen's health needs becomes more important as health complexity increases. Health complexity in turn drives choices around the types of virtual care technologies that will be used. On the y axis, technology complexity ranges from low to high. Technological evolution has enabled the use of virtual care technologies that range from the simple (low complexity) to elaborate (highly complex) for patient diseases and conditions that are low to highly complex. In every case the precision of technology fit with patient health complexity determines patient outcomes (see Table 2 for examples).

**Table 2.** Patient Complexity and Technology.

Patient Complexity	Technology Complexity	Healthcare Example
Low	Low	Use of portals during the COVID-19 pandemic to reduce social isolation and remain informed about the current health recommendations [ 10 ]
Low	High	Tele-dermatology and dermatology home consultations are done in conjunction with telemedicine services [ 34 ]
High	Low	Hospital at Home [ 35 , 36 ]
High	High	Tele-ICU [ 37 ]

To illustrate the authors will provide several virtual care examples, where patient contexts and technology, are considered in terms of complexity as a way of identifying candidate technologies that can provide precision (i.e., tailored) healthcare.

### 4.1. Low Patient Complexity and Low Technology Complexity

In the lower left-hand corner of Figure 1 , citizens, who are relatively healthy may use a widely available, easy to use technologies. This might include technology such as a web portal to obtain information about wellness. Senior citizens (i.e., those over the age of 65) are a rapidly growing segment of society that use technologies for health and wellness. In Sweden during the early part of the recent COVID-19 pandemic, when vaccines and treatments were still being developed and tested, many seniors voluntarily quarantined themselves to avoid contracting and spreading COVID-19 [ 37 ]. To address their social and health-related information needs, many seniors used varying technologies to connect with friends and family, to take part in social-cultural activities, and to stay up to date on the latest health information via official health portals.

Here, virtual care technologies such as the Internet and citizen portals were used to maintain wellness and social contacts while at the same time reducing feelings of isolation and loneliness [ 10 ]. Portals, and websites were created, or existing patient portals were modified by governments and healthcare organizations as a way of communicating information about COVID-19 to influence public, health and wellness behaviours.

These technologies are increasingly being designed with simple and easy to use interfaces with an emphasis on usability and usefulness of information [ 38 ]. Unlike technologies developed for individuals with complex health conditions and diseases, these technologies may be used to provide general information that can be used by anyone, who is healthy or is self-managing a chronic illness, so technology complexity is low (i.e., a website or portal) in terms of access to usable and useful information [ 39 ]. A website or portal provides information to a wide range of individuals, who are living in the community, are healthy and/or are successfully managing their health conditions or chronic illnesses. A recent systematic review reported that seniors are interested in specific features of patient portals that would help them self-manage their health [ 40 ]. Websites and portals are technologies that are frequently used by citizens to obtain health information to maintain wellness. The usability, usefulness and commonality of these sites makes this technology of low complexity. Along these lines, the well elderly and those who can effectively self-manage their health conditions are of low complexity.

### 4.2. Low Patient Complexity and High Technology Complexity

In the upper left-hand corner of the framework, citizen health conditions and diseases may be low in complexity but may still require the use of complex virtual care technologies. To illustrate tele-dermatology and dermatology home consultations are done in conjunction with telemedicine services (in the Netherlands). These services facilitate digital dermatological healthcare to diagnose and manage a single skin condition/lesion of a patient through a virtual platform. This eliminates the need of patients to physically visit a dermatologist. Tele-dermatology, digital consultation about a patient's skin condition takes place between a primary care provider (general practitioner) and a secondary care provider (dermatologist) and this represents a complex configuration of several technologies. Tele-dermatology is mostly done in a store-and-forward manner, where the sending and receiving healthcare practitioner are independent of time and location since no live communication is needed. Further, there are also digital home consultation services implemented, where a patient can digitally send their taken photos with their own smartphone to their general practitioner via a secured service. Again, without the need to physically visit the GP with the corresponding travel and waiting time [ 41 ]. These two types of tele-dermatology services are perfectly suitable for patients with low complexity, skin conditions, where one type of skin condition/lesion is presented that needs further investigation.

Both tele-dermatology and patient home consultation services require readily available technologies, such as an internet connection to access a virtual platform to send the consultations, and smartphones and camera equipment (e.g., digital dermoscope) to take the digital (dermoscopic) images. To use these technologies, practitioners and patients require some limited technical and photography skills. Once these skills and requirements are in place, a consultation can be easily sent by a general practitioner and responded to by a dermatologist, even within only a few minutes. But one might discuss that not all patients and practitioners (especially the elderly or untrained) have adequate health literacy to use these services. However, the underlying basics of these skills (i.e., use of a smartphone/camera to take images and the Internet) belong to the daily habits of citizens nowadays, and the widespread use of smartphones and the Internet in daily life emphasizes the relative simplicity of these technologies. This makes it accessible for a wide range of individuals. Thus, tele-dermatology not only addresses low patient health complexity by requesting advice for one single skin lesion, but also using several technologies by using an online virtual platform so that the tele-dermatology services are accessible is a highly complex activity for all [ 34 ].

### 4.3. High Patient Complexity and Low Technology Complexity

In the lower right-hand corner of Figure 1 , we have an example of patients, who are highly complex and the technology that is used to care for them is of low complexity. Patients, who are highly complex (i.e., they may have a medical condition that requires attention, real-time monitoring, and/or an admission to the hospital), but can be sent home with health professional monitoring and technology supports is an example of high patient complexity and low technology complexity. This model of care is sometimes called Hospital at Home. Hospital at Home is being used as an alternative to admitting patients to hospital. In a Hospital at Home model of care, patients are registered or admitted to hospital, but they receive healthcare at home. Patients receive frequent in-person and virtual visits with the Hospital at Home physician and health professional team due to the acute nature of their condition. The use of in-person and virtual visits allows for the patient (and their family) to have access to health professional team at any time (i.e., throughout a 24-hour day). Patients are provided with most therapies, medications, tests, and vital signs monitoring in the home [ 42 , 43 ]. Technology supports involve the use of widely used medical devices (e.g., blood pressure monitor) and communication tools (e.g., video conferencing calls) [ 44 ]. However, patients may receive more involved testing such as x-rays or CT scans that still require a physical appointment at the hospital) [ 35 , 36 ]. Hospital at Home is an example of high patient complexity and low technology complexity used to care for individuals in their homes. Patients are highly complex because their medical condition requires immediate attention and real-time monitoring by health professionals using technologies. The technologies that are used to monitor the patient may be of low technology complexity (e.g., blood pressure monitor, oxygen monitoring device) as they are commonly used by health professionals and can be effectively used in the home.

### 4.4. High Patient Complexity and High Technology Complexity

In the upper right-hand corner of the framework, we describe an example, where patient and technology complexity are both high. In this example we discuss the Tele-ICU, a virtual care technology that allows patients to be cared for by health professionals from a distance. Tele-intensive care or Tele-ICU refers to the monitoring of remotely located ICU patients to detect deterioration and/or to provide consultation to ICU patient health professional caregivers. The Tele-ICU consists of real-time, remote access to patient (citizen) data. The data is drawn from physiologic monitors, ventilators, laboratory tests, radiographic images, medication records, clinical documentation and ePrescribing systems. Tele-ICU patients are managed via complex decision support systems that use algorithms to provide alerts and alarms. Health professional teams in the Tele-ICU review patient data and work with care team members who are providing direct patient care to the patient. When alerts or alarms occur, members of the Tele-ICU team such as physicians communicate directly with health professionals in the ICU via video communications. Tele-ICU's help bring members of the health professional team together (i.e., those health professionals that are working remotely and those that are providing care to the patient) thereby providing direct care providers with access to highly qualified critical care physicians and nurses at a distance. Acute health crises require the involvement of Tele-ICU technologies. Here, such technologies help to manage hospitalized patients (the highly complex) and to connect health professional caregivers using highly complex technologies that support life.

## 5. Conclusion and Future Research Directions

Several factors require consideration, when designing virtual care systems, to provide precision (tailored) medicine to citizens (and patients). With technological advances, the number of virtual care opportunities to provide precision patient care have expanded [ 44 ]. Researchers have proposed evidence-based approaches and methods to determine the types of technologies that best fit the health needs of individuals. In this paper we describe a framework for considering and reasoning about Precision Virtual care. The framework considers the relation between patient (or citizen) complexity and technology complexity. We have also identified several examples of varying complex technologies that can be used to address aspects of patient complexity. Given the technological advances that have occurred recently and our ability to create digital ecosystems of care, we are about to embark on a new era of patient and technology driven precision, virtual care. There is a need for support in reasoning about technology-patient fit to ultimately lead to precision virtual care. The work in this paper represents and initial approach for doing so and could be expanded to include additional dimensions for considering key aspects such as patient e-health literacy when deciding on the match between technology and the patient context (e.g., when implementing as well as procuring virtual care solutions).

Technolog(ies) and digital health ecosystems of care will need to be designed and implemented to address patient complexity. Some of this work will include developing a better understanding of patient populations including their diseases, disease trajectories and health conditions. Patient's and healthcare professionals will also need to be studied in terms of their digital health literacies and the associated impacts of such knowledge on their ability to understand and use technology to maintain wellness, self-manage disease(s), and participate in the process of regaining or restoring health. Critical to this work will be identification and selection of metrics that can be used to evaluate the impacts of precision or tailored technologies upon the health of citizens. Such work will be critical to determining the future of virtual care and comparing its efficacy to other more traditional approaches to providing care. Lastly, it must be noted that virtual care technologies may be tailored to individual patients and their associated diseases and health conditions, but that in some cases face-to-face visits with a health professionals may still remain the best approach to receiving healthcare.
